# Activities of the healthcare team for women who smoke during pregnancy and the puerperium^1^

**DOI:** 10.1590/0104-1169.3361.2460

**Published:** 2014

**Authors:** Carolina de Castilhos Teixeira, Amália de Fátima Lucena, Isabel Cristina Echer

**Affiliations:** 2 Master's student, Escola de Enfermagem, Universidade Federal do Rio Grande do Sul, Porto Alegre, RS, Brazil; 3 PhD, Adjunct Professor, Escola de Enfermagem, Universidade Federal do Rio Grande do Sul, Porto Alegre, RS, Brazil

**Keywords:** Tobacco Use Cessation, Patient Care Team, Health Education

## Abstract

**OBJECTIVE:**

to identify activities developed by the healthcare team for pregnant and postpartum women who smoke.

**METHOD:**

cross-sectional study with a sample of 135 healthcare team members who assist pregnant and postpartum women in a university hospital located in southern Brazil. The data was collected using questionnaires and analyzed using the Statistical Package for Social Sciences software.

**RESULTS:**

76 (56.3%) staff members reported that they always addressed smoking cessation; however, the approach occurred in only two periods of the hospitalization and/or prenatal consultations, not including family members. In regard to the effectiveness of their actions, the health team assessed it as fair or poor, and mentioned the need for updating knowledge regarding this issue.

**CONCLUSIONS:**

the health team did not perform the approach as recommended by the tobacco control guidelines, requiring training to offer a qualified and efficient intervention.

## Introduction

In Brazil, on average, 1,600 women die each year due to pregnancy, childbirth and postpartum issues and 50,000 children die under the age of one year. Among the causes are the complications associated with use of substances during pregnancy, including nicotine^(^ 1 ^)^.

Studies from Southern Brazil revealed a prevalence of smoking among pregnant women from 23% to 27.5%, high rates when compared to the adult female population, which is 15.9%. Pregnant smokers also have lower education levels and conduct fewer prenatal visits than non-smokers. Among the smokers studied, only 1.19% received some type of care/medication treatment for smoking cessation^(^ 2 ^-^ 3 ^)^.

Considering these data and given that the period of pregnancy, childbirth and postpartum is a moment when a woman is more likely to interrupt her smoking behavior, because the baby motivates her to look after her own health and she starts to have more contact with healthcare professionals^(^ 4 ^)^, the performance of cognitive-behavioral approach is recommended for pregnant and postpartum smokers. This approach consists of preparing them to address their problems and resist smoking; preventing relapse and coping with stress; and, in cases of high dependence on nicotine, providing medication therapy support. The cognitive-behavioral approach is considered the basis of the intervention, essential for smoking cessation, with data suggesting a cessation rate directly proportional to the time devoted to this approach^(^ 5 ^-^ 6 ^)^.

One systematic review showed that health professionals used standardized instruments to identify the smoking profile, nicotine dependence, craving, abstinence and motivation for cessation, in order to help them to intervene in the health education process in these patients^(^ 7 ^)^.

On the other hand, a study conducted in Southern Brazil, with medical and surgical patients, showed that actions performed by nurses were aimed toward the rules guiding the prohibiting of smoking in the institution, and about the possibility of smoking cessation without the use of specific instruments or cognitive-behavioral approach^(^ 8 ^)^, a fact that identifies knowledge gaps on this subject.

Thus, considering the need to improve the actions for smoking cessation and the situation of pregnancy/postpartum, which may trigger a positive motivation for this, the present study aimed to identify the activities developed by the healthcare team for pregnant and postpartum smokers. To do so, we defined as actions of the healthcare team every activity, intervention and guidance focused on health promotion in relation to smoking cessation for this population

## Methods

This was a cross-sectional study, conducted in a university hospital in southern Brazil. The population consisted of multidisciplinary team members of an obstetric outpatient unit and a pre-and postpartum hospital unit, assisting pregnant and postpartum women. The sample was defined by convenience, not proportionally, and was composed of physicians, nurses, nutritionists, psychologists, pharmacists, social workers, nursing assistant staff, residents and students who agreed to participate.

The sample size was calculated based on a study that showed a prevalence of 61.5% of professionals addressing smoking issues^(^ 9 ^)^ during patient care. Considering a confidence interval of 95% and an acceptable error of 10%, we calculated a sample size of 91 participants. However, we invited all 170 members of the health teams in the selected units that met the inclusion criteria, with 135 of them agreeing to participate and being included in the present study sample.

The inclusion criteria were: being part of the healthcare team of the units assisting pregnant and postpartum women during the period of data collection, and being students in the second semester of the course or above.

Data collection was conducted using a non-validated questionnaire, developed by the researchers, which included questions about education and smoking status of the participants, and activities developed with smokers and their efficacy. A final open-ended question requested suggestions for approaches or actions to be performed with the target audience. The instrument was given to the participants during their working hours in the months of August and September of 2012 and collected in a sealed envelope, in order to preserve the identity of respondents.

Data were entered and organized in a database and analyzed using the *Statistical Package for Social Sciences* (SPSS), version 18. Descriptive analysis of absolute and relative frequency, mean, median, standard deviation and percentiles were performed. For categorical and continuous variables, the following tests were performed: Fisher's exact test, Chi-square test, Mann-Whitney and Kruskal-Wallis test. Every association and every difference with a p-value of less than 0.05 was considered statistically significant. The open-ended question was analyzed quantitatively; the responses were grouped by the content addressed.

The project was approved by the Ethics Committee of the institution under CAAE number 05182112.2.0000.5327, and all participants signed the terms of free and informed consent form.

## Results

A total of 135 members of the healthcare team participated in the survey. Their characteristics are presented in Table 1.

**Table 1:** Characteristics of the members of the healthcare team in obstetrical areas, Porto Alegre, RS, Brazil, 2013

Sample profile		n (%)
Age		30 [25-49]*
Sex		
	Female	102 (75.6)
	Male	33 (24.4)
Civil status		
	Single	66 (48.9)
	Married/Stable relationship	55 (40.7)
	Divorced	12 (8.9)
	Widowed	2 (1.5)
Schooling in years		17 [14.2-18.5]*
Profession		
	Student	49 (36.3)
	Nursing assistant	37 (27.4)
	Medical doctor	23 (17.0)
	Nurse	17 (12.6)
	Resident doctor	5 (3.7)
	Nutritionist	2 (1.5)
	Social worker	1 (0.7)
	Pharmacist	1 (0.7)
Work years in the health sector		10 [5-21]*
Work years in the obstetric field		3 [0.5-15]*

* Median in years

Among the students, 45 (91.8%) had already completed more than half of their undergraduate course, 15 (30.6%) were in the 8^th^ semester of nursing, medicine or psychology, 11 (24.4%) were enrolled in the 12^th^ semester of medical school and 8 (17.8%) were enrolled in the 11^th^ semester.

A significant association (p>0.001) was observed when comparing the mean age of smokers, abstinent smokers and nonsmokers. The median age among groups showed a significant age difference in which the group of smokers and abstinent smokers presented a value of 54 years [IQ=34 to 58.5], while in the group of non-smokers it was 28 years [IQ=25-42].

In regard to the approach that the staff used on health issues related to smoking, 76 (56.3%) healthcare team members mentioned always doing it, 46 (34.1%) sometimes, and 13 (9.6%) did not approach these issues with their patients. In 92 (78.6%) of the cases, the approach occurred twice during hospitalization or during the prenatal consultations, and in 25 (21.4%) observations it occurred three or more times; 62 (45.9%) approaches did not include family members, 53 (39.3%).

By analyzing the approach performed by the different categories of health staff in relation to smoking, physicians and nurses stood out with the highest percentage of "always performing the approach". This analysis was performed only with members that showed an n≥10, in order to preserve the identity of the participants (Table 2).

**Table 2:** Approach of the healthcare team in relation to pregnant and postpartum smokers, Porto Alegre, RS, Brazil, 2013

Approaches		Healthcare team members										
		Medical doctors (N=23)			Nurses (N=17)			Students (N=49)			Nurse assistants (N=37)	
		n	%		n	%		n	%		n	%
Approach smoking issues												
	Always	17	73.9		12	70.6		31	64.6		13	35.1
	Sometimes	5	21.7		5	29.4		14	29.2		17	45.9
	No	1	4.3		0	0		3	6.3		7	18.9
Number of times												
	Once	12	52.2		8	47.1		32	66.7		18	48.6
	Twice	0	0		4	23.5		7	14.6		4	10.8
	3-4 times	5	21.7		2	11.8		2	4.2		2	5.4
	>4 times	5	21.7		3	17.6		4	8.3		1	2.7
Approach family												
	Yes	5	21.7		3	17		6	12.5		6	16.2
	Sometimes	8	34.8		9	52.9		21	43.8		11	29.7
	No	10	43.5		5	29.4		21	43.8		20	54.1

The association between "address the patient about tobacco issues" and the different team members was statistically significant (p=0.025), as well as the association of "number of approaches" and "team members" (p=0.008). The residual analysis showed that the nursing staff made fewer approaches than expected for each category (Table 2). 

When participants were asked about their attitude towards a pregnant or postpartum woman who was resistant to smoking cessation, we obtained the following **RESULTS:** 102 (80.3%) subjects stated that they reinforced guidance to patients about the harm that tobacco can cause to the health of both mother and baby, as well as the benefits associated with its cessation; 15 (11.8%) reported not pursuing the matter and preferring to wait for a new appointment to return to discussion of the problem; 13 (10.2%) preferred to respect the choice of the patient about smoking and did not further address this issue; and 10 (7.9%) used shocking ideas, trying to raise awareness by patients to abandon smoking behavior through the use of fear and pressure.

**Figure 1:**
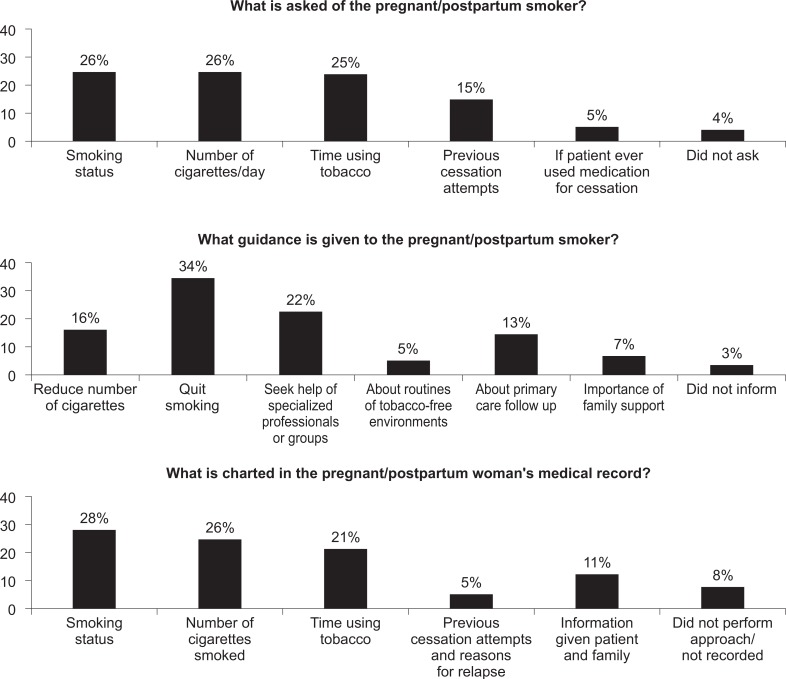
Investigation, guidance and medical records of the healthcare team when assisting pregnant and postpartum smokers, Porto Alegre, RS, Brazil, 2013

The healthcare team investigations of pregnant or postpartum smokers were mainly focused on smoking status [94 (26%) cases], the number of cigarettes smoked per day [93 (2.6%) cases], and the time using tobacco [90 (25%) cases]. The health team responded that guidance for patients to quit smoking was given 103 (34.4%) times; to seek help from professionals/groups was suggested 65 (22%) times; and to reduce the number of cigarettes smoked was reported 47 (16%) times. As for the record in the medical charts, 89 (28.3%) responded that data were related to the smoking status of the patients, 82 (26%) to the number of cigarettes smoked per day, and 67 (21.3%) to the time using tobacco (Figure 1).

Regarding the use of tools that assisted interventions regarding smoking behavior, 103 (82.4%) subjects reported no use of any instrument, 17 (13.6%) performed the cognitive-behavioral approach, 5 (4%) used the Fagerström scale and 3 (2.4%) evaluated the motivation level as recommended by Proshaska and Di Clemente. In only 8 (17%) cases, academics, the application of the Fargerström Scale and the Stages of Motivation for Smoking Cessation Scale were reported. 

Among the barriers that hindered intervention in the process of tobacco cessation, 73 (61.9%) participants indicated the lack of time during consultations, 32 (27.1%) few institutional incentives, 27 (22.9%) the lack of skill and knowledge about smoking cessation, and 23 (19.5%) the unavailability of specific treatments; 20 (16.9%) participants indicated other impediments, such as the brief bond with the hospitalized patient, the resistance of patients to this approach, and forgetting to do it.

Fifty (38.8%) participants said they felt difficulties with informing pregnant and postpartum women regarding smoking cessation. Seventy-three (57%) stated that the team's activities sometimes contributed to smoking cessation among pregnant and postpartum women, while 30 (23.4%) believed that they "always" contributed, and 25 (19.5%) felt that they did "not" contribute.

Ninety (70.3%) participants rated the efficacy of their actions for the cessation process as fair, 29 (22.7%) as poor, and 9 (7%) as excellent or good. For 67 (51.1%) of the participants, the discussion of the theme "tobacco and pregnancy" did not occur in the workplace, and 105 (81.4%) reported feeling the need to update or deepen their knowledge in this area to improve their care.

Thirty-six (26.7%) participants suggested actions that the health team could put into practice to assist pregnant and postpartum women in modifying their smoking behavior. Of these, 21 (58.3%) reported preventive actions, guidelines and approaches in the primary care level, educational activities for injury reduction, and production of lectures and informative materials; 10 (27.8%) suggested the formation of a multidisciplinary team specialized in the care of these patients; 6 (16.7%) proposed intensifying training for the multidisciplinary team; and 3 (8.3%) recommended the formation of more groups for smokers to attend, and the availability of more medications to aid cessation. The reported values exceed 100% because some participants suggested more than one action.

## Discussion

Healthcare team activities have been partially developed without systematic application of scales/instruments or the use of the cognitive-behavioral approach. The results show a reality distant from what is recommended by the Ministry of Health, which suggests the use of a cognitive-behavioral approach with pregnant and postpartum women based on the initial assessment of patient dependence, and medication therapy in specific situations^(^ 5 ^)^.

In another study, the guidelines of the Ministry of Health regarding the approach to smoking patients were partially followed by slightly more than half of the sample^(^ 5 ^)^. The unsatisfactory approach observed is reflected in the results of the healthcare team self-assessment in relation to cessation, in which more than 90% rated their action as fair or poor. This highlights the need to review the training of teams to better approach patients and their families.

Medical doctors and nurses were the ones who more often provided assistance to the smoking patient, including the family in this care. This finding is consistent with a study that relates these professionals and the patients' relatives as the major sources of patient interventions and guidelines when it comes to smoking^(^ 4 ^)^. However, it is known that for the behavior change to occur, much more than two interventions are required; so it is imperative that they occur in a systematic way to actually help this group of patients to quit smoking. Thus, the results point to the need of the healthcare team to review and enhance approaches to the patients and their families.

The nurse assistant staff had the lowest number of approaches and they reported having more difficulty in providing guidance activities, which can be explained by the fact that their training is often focused on developing skills to perform specific tasks and procedures^(^ 10 ^)^. Taking into consideration that this is the largest professional category in hospital care, and those who have the greatest contact with patients in this setting, the development of training strategies is suggested to enable them to change this reality.

One study showed that devaluation or omission of actions by the health professionals regarding smoking behaviors may be interpreted by the patient/family as it having no relationship to their disease process and recovery^(^ 11 ^)^. Another study^(^ 12 ^)^ showed that a brief intervention by the health team could be decisive for the patient decision to quit smoking. Therefore, it is necessary that the interventions occur during all favorable opportunities and by all members of the healthcare team.

The questions, guidelines and medical records were mainly focused on the identification of smoking status, its characteristics, and information provided for tobacco cessation. Such data is essential in the context of the cessation process, since the choice of the most appropriate treatment is developed based on these. However, for cessation to occur, the mere collection of data is not enough; the monitoring of patients is necessary, as is the performance of interventions during hospitalizations or in the prenatal consultations, using the cognitive behavioral approach and medications, when necessary.

Counseling was conducted briefly, however the results reinforced the need for guidance to be carried out systematically according to the level of understanding of the patients, so it can be successful. Studies^(4,13) ^showed that pregnant and postpartum smokers had lower educational levels, and some did not fully believe in the harm that tobacco causes to the baby. They also presented a lack of social support and were subjected to stress, which reinforced the need for the support of the staff in this process.

The Fagerström and Motivation scales were used only by medical and nursing students, which may be related to the fact that such instruments are not part of most professionals' field of knowledge^(^ 14 ^)^. Based on this result, we suggest the training of health teams by distributing these instruments, which assist in driving the choice of the best treatment.

The most commonly performed activity for smoking patients who did not want to quit smoking was providing information about the harm that it can cause to the mother and baby, and the benefits of cessation. This approach meets the Ministry of Health guidelines, which recommend a review of the patient's motivation to stop smoking, or not, and conducting motivational interventions^(^ 5 ^)^. This approach is essential to help pregnant and postpartum smokers to think about the risks of continuing smoking, and the benefits of quitting.

The prevalence of smoking among health team members was low when compared to the general population, which was 15%^(^ 5 ^)^. The smokers and abstinent ones had statistically higher mean ages than non-smokers, which can be explained by the fact that, over the past three decades, with legislation and the support of cessation programs, there was a decrease in tobacco use throughout the country^(^ 15 ^)^. An international study identified a prevalence of 33% of smokers among nursing students, and highlighted the need to discourage tobacco consumption and work toward implementation of programs to train health professionals in tobacco control and effective cessation counseling techniques^(^ 16 ^)^. The results of this study show that there is also the need for constant training of the teams on theoretical and/or practical aspects involved in smoking cessation, beginning in the undergraduate period.

The barriers identified by health staff for the tobacco cessation process are worrisome, since a review study^(^ 13 ^)^ showed that the best time to approach these patients and to obtain better results is during the prenatal consultation, when it is possible to achieve cessation rates up to 70%. In addition, over 80% of the staff members mentioned an interest in updating and improving their knowledge in the area, in order to better assist these patients. Therefore, it is urgent that health teams are trained to act with prevention, guidance and approaches to prevent the initiation of tobacco beginning in childhood. Specialized groups in smoking cessation are capable of better assessment, treatment and monitoring of tobacco users, bringing the multi-professional actions closer to what is recommended by the National Guidelines for Tobacco Control^(^ 6 ^,^ 17 ^)^.

Despite this fact, the results achieved in primary care in smoking cessation programs were around 35%^(^ 17 ^)^, higher than those reported in the literature, which reinforced the importance of the approach with any contact with the smoking patient, both during consultation in primary care and hospitalization. These moments are characterized by the possible presence of diseases related to smoking behavior, constituting a favorable opportunity to change the habit of the smoker^(^ 4 ^,^ 11 ^)^.

The results of this study showed the need for smoking to be assessed, monitored and treated by the healthcare team daily, with appropriate interventions in supportive and/or necessary moments. However, the staff needs to be trained for this and it is up to the institution to develop policies and protocols covering treatment and care for smokers, especially for pregnant and postpartum women.

This study had some limitations, such as the fact that the team members responded to a printed questionnaire, which may have led to different interpretations of the questions, and the fact that they were responding about their own actions with risk of bias related to overestimation of their actions. However, it is understood that the process of cessation, which for some people is long, difficult and includes relapses, requires a healthy relationship between the professional and the patient to succeed. For this to happen, knowledge, availability and resolve are required of the healthcare team, which can facilitate the process of tobacco cessation among patients. 

## Conclusions

The recommended actions for the care of pregnant and postpartum women who smoke were being partly developed by the healthcare members studied, predominantly by physicians and nurses. This approach occurred only in two occasions during hospital admissions or pregnant/postpartum consults, and did not include family members. Health teams did not use assessment instruments with the smoking patient, and questions, guidelines and records appeared to be exclusively devoted to the collection of information on tobacco use, with the interventions being incipient. Therefore, we concluded that a change in the approach to the smoking patient was imperative, with reorganization of health teams to develop actions to prevent and treat tobacco use. Despite being aware of the problem, team members reported that they knew little about the subject and did not have time in clinical practice to assist with this serious public health problem. It is understood that there is a need to stimulate health teams to systematically and respectfully address pregnant and postpartum women, aiming toward the process of smoking cessation. For this, we need to empower them not only for the correct handling of medication therapies, but also for the proper guidance about the harms of smoking, with the development of skills and techniques capable of breaking the barriers related to lack of time and misinformation, to help these women to quit smoking and remain abstinent. Therefore, the intervention of the healthcare team, even minimal, can help change the course of this history and make a difference to the quality of health of the mother/baby.
